# P-577. Clinical Predictors of Mortality in Hospitalized Patients with Rickettsioses in Northern Mexico

**DOI:** 10.1093/ofid/ofaf695.791

**Published:** 2026-01-11

**Authors:** Karla Cuellar-Calderon, Paola Bocanegra-Ibarias, Samantha Perez-Cavazos, Perla Gonzalez-Vazquez, Magaly Padilla-Orozco, Adrian Camacho-Ortiz

**Affiliations:** Servicio de Infectologia - Hospital Universitario " Dr. Jose Eleuterio Gonzalez", Monterrey, Nuevo Leon, Mexico; Servicio de Infectologia - Hospital Universitario " Dr. Jose Eleuterio Gonzalez", Monterrey, Nuevo Leon, Mexico; Servicio de Infectologia - Hospital Universitario " Dr. Jose Eleuterio Gonzalez", Monterrey, Nuevo Leon, Mexico; Servicio de Infectologia - Hospital Universitario " Dr. Jose Eleuterio Gonzalez", Monterrey, Nuevo Leon, Mexico; Servicio de Infectologia - Hospital Universitario " Dr. Jose Eleuterio Gonzalez", Monterrey, Nuevo Leon, Mexico; Universidad Autónoma de Nuevo León, Monterrey, Nuevo Leon, Mexico

## Abstract

**Background:**

Rickettsioses are life-threatening vector-borne infections^1^. Clinical manifestations are nonspecific, commonly including fever, malaise, and an exanthematous rash, necessitating a high index of suspicion for timely diagnosis. Without appropriate treatment, mortality can reach 100%^2^.

**Figure:**
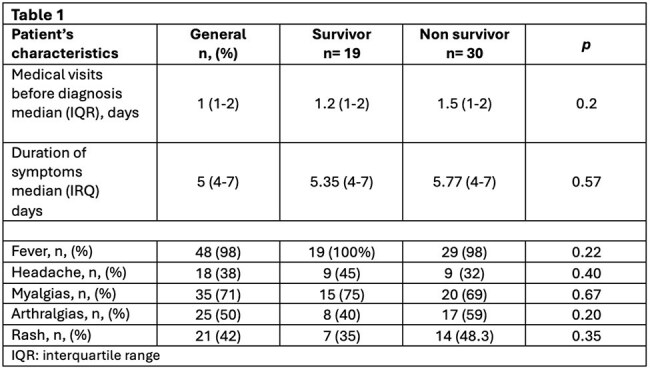

**Figure:**
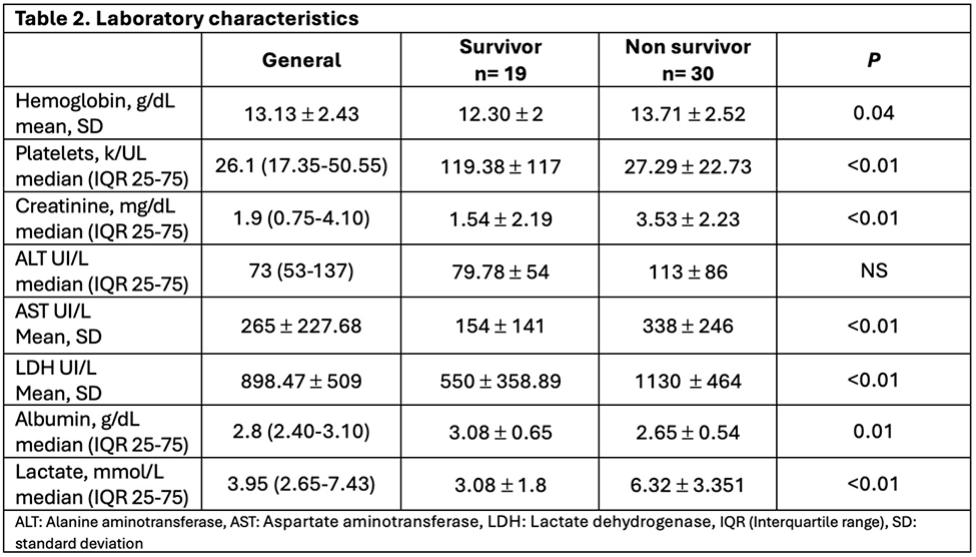

**Methods:**

From October 2022 to April 2025, we conducted a prospective study of patients with qPCR-confirmed rickettsioses at a tertiary teaching hospital in Monterrey, Mexico. Data were analyzed using SPSS v19. We applied Student’s t-test and chi-square test for group comparisons and calculated relative risks (RR).

**Figure:**
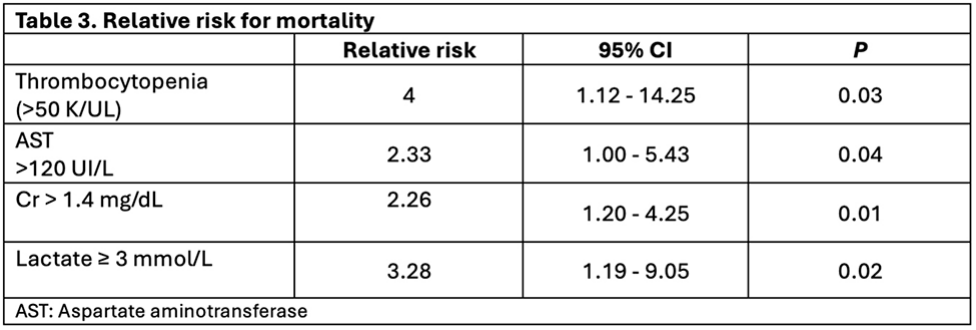

**Results:**

A total of 49 patients were included, with a median age of 15 years (range 9-32 years), and 52% were males. Thirty patients (60%) died during hospitalization.

The media time-to-treatment from hospital admission was 5.15 hours. All patients received doxycycline. Some patients received antibiotics prior to admission, including ceftriaxone and amoxicillin/clavulanic acid; only three received doxycycline.

The median number of medical visits before diagnosis was 1 day (range 1-2)

Symptom duration before admission had a median of 5 days (range 4-7). Fever (98%) and petechial rash (42%) were the most common clinical signs shown in Table 1, while thrombocytopenia was present in 98% of patients.

Factors associated with increased mortality were thrombocytopenia RR 4 (95% CI 1.12-14.25) p = 0.03, elevated lactate RR 3.28 (95% CI 1.19.9.95) p = 0.02, increased creatinine RR 2.26 (95% CI 1.2-4.25) p = 0.01 and aspartate aminotransferase (AST) levels RR 2.33 (95% CI 1-5.43) p = 0.03, as shown in Table 3.

**Conclusion:**

Rickettsioses are a critical illness associated with high mortality. In our cohort, fever was a common presenting symptom, while rash was observed in fewer than half of the patients; notably, 98% of the patients presented with thrombocytopenia. Elevated lactate levels, acute kidney injury, and thrombocytopenia at admission were significant predictors of mortality.

**Disclosures:**

All Authors: No reported disclosures

